# Should Malaysia Widen Patient Access to Medical Records? An Ethical Analysis

**DOI:** 10.1007/s41649-025-00359-z

**Published:** 2025-08-28

**Authors:** Astrid Sinarti Hassan

**Affiliations:** Department of Medical Ethics and Law, Faculty of Medicine, Universiti Teknologi MARA, Kuala Lumpur, Malaysia

**Keywords:** Deontology, Malaysia, Moral theories, Patient access, Medical records, Utilitarian

## Abstract

This paper examines the ethical justification for wider patient access to their own medical records, focusing on utilitarian and deontological perspectives. At present, despite acknowledging that patients have rights to their medical records in Malaysia, this access is restricted. The utilitarian approach, rooted in consequentialist theory, advocates for actions that produce the greatest benefit for most people. It emphasises that patient access to medical records can enhance patient empowerment, engagement and overall health outcomes. Despite concerns from healthcare providers about potential patient confusion and misinterpretation, empirical studies indicate that patients generally benefit from access, feeling more in control and involved in their healthcare decisions. Conversely, Kant’s deontological theory prioritises the morality of actions based on their inherent nature rather than outcomes. It supports unrestricted patient access as a moral obligation, emphasising respect for patient autonomy and truth-telling. According to this theory, withholding information from competent patients is ethically unjustifiable and infringes on their rights. Both ethical frameworks reveal significant patient benefits and challenge the traditional paternalistic approach of healthcare providers. This paper concludes that wider patient access to medical records in Malaysia is ethically justifiable and beneficial, urging legal and ethical reforms to support this practice.

## Introduction

In modern bioethics and law, the concept of autonomy occupies a central role, reflecting a profound shift in the way patients are viewed within healthcare systems. Autonomy, in its most fundamental sense, refers to the right of individuals to make informed, uncoerced decisions regarding their own lives, particularly their healthcare choices (Beauchamp and Childress 2013). This principle has evolved within both legal frameworks and ethical philosophies, influencing policies and practices related to medical decision-making. Notably, the Oviedo Convention (Council of Europe 1997), which articulates the protection of human rights in the biomedical field, underscores the importance of autonomy as a foundational element in safeguarding individuals’ dignity and personal integrity in healthcare.

The philosophical underpinnings of autonomy are grounded in two predominant schools of thought: deontology and utilitarianism. These frameworks offer differing perspectives on the role and limits of autonomy in decision-making. Deontological ethics, particularly as articulated by Immanuel Kant, holds that individuals possess intrinsic dignity and must be treated as ends in themselves, not merely as means to an end (Kranak 2019). This view emphasises the fundamental right of patients to access their medical records, as doing so enables informed choices and respects their inherent autonomy. By contrast, utilitarianism focuses on the outcomes of actions, suggesting that restrictions or non-restrictions on access to medical records might be justified if they lead to greater societal or individual benefit.

In the legal context, respect for autonomy has become a cornerstone of patient rights, particularly regarding access to medical records. The modern legal landscape increasingly recognises the importance of ensuring that patients can not only make informed decisions about their treatment but also have the necessary tools such as complete access to medical records to do so effectively. The Oviedo Convention reflects this shift, emphasising the need for transparency in all matters relating to healthcare, while also recognising the need for balancing autonomy with considerations of privacy and confidentiality (Council of Europe 1997).

In Malaysia, the Malaysian Medical Council (MMC) recognises a patient’s right to access medical records (Malaysian Medical Council 2006). However, regulation on this issue is inconsistent, often requiring a court order for patients to gain access.^1^ This regulatory complexity fosters practices and values such as medical paternalism and therapeutic privilege, compounded by the high status that doctors enjoy in the doctor-patient relationship (Lipworth et al. 2013). While there is no empirical evidence explicitly demonstrating this effect, there is limited discussion on issues such as patient rights to access their medical records in Malaysia.

In contrast, patient access to medical records is well-established in healthcare systems such as that of the UK, where patients’ rights are typically balanced against privacy concerns, but with greater transparency and autonomy in practice. This article seeks to fill a significant gap in scholarship by examining the regulatory complexities surrounding patient access to medical records in Malaysia, using moral theories to explore whether the expansion of these rights is warranted. By scrutinising the challenges of balancing autonomy with the practical realities of healthcare regulation, this work aims to assess whether Malaysia should reform its policies to align more closely with international norms.

## Ethical Foundations: Deontology and Utilitarianism

Both frameworks, while differing in their approaches, ultimately converge in advocating patient access to medical records. The choice to focus on deontology and utilitarianism in this discussion arises from their prominence as two of the most influential ethical frameworks in bioethics and medical law. These schools of thought allow for a robust analysis of patient access to medical records from distinct perspectives, ensuring a well-rounded exploration of the issue. Utilitarianism introduces a more pragmatic approach that considers the broader consequences of granting or restricting access to medical records. Although utilitarianism acknowledges the importance of patient autonomy, it requires that such autonomy be weighed against the potential harms and benefits to the individual and society. This framework allows for an examination of the potential trade-offs involved, such as the risks to privacy and the potential for psychological harm from exposure to sensitive medical information. However, even within this consequentialist framework, the goal remains the same: maximising the well-being of patients through informed decision-making.

Deontology, with its emphasis on rights and duties, provides a clear moral imperative that supports patient access to their records as a matter of justice and respect for autonomy. By framing the issue through a deontological lens, we can address the argument that denying patients access to their medical information undermines their ability to make informed decisions, thus violating their moral rights.

Ultimately, both deontology and utilitarianism, despite their contrasting approaches, converge on the conclusion that patient access to medical records is ethically justified through empirical research that offers critical insights into the practical implications of such access. Empirical studies may shed light on the actual benefits and challenges faced by patients and healthcare providers, providing a more nuanced understanding of the ethical justification for expanding patient access to their medical records in Malaysia.

## Empirical Evidence on Patient Access to Medical Records

Although the previously discussed legal and philosophical viewpoints establish a solid theoretical basis for patient access to medical records, empirical research provides important insights into the practical outcomes of such access. By examining how patients and medical professionals interpret and respond to the concept of viewing their own medical information, these studies offer valuable information that helps quantify the potential benefits and harms of such access.

Empirical evidence supports patient access to medical records, despite varying concerns from healthcare providers. A study examined cancer patients’ reactions and doctors’ preconceptions regarding patient access to medical records using semi-structured interviews (Fisher and Britten 1993). The results showed that patients who chose to look at the records found them helpful and reassuring, despite information about a bad prognosis. Doctors expected that access would cause harm to patients but did not wish to amend the entry. The majority of doctors expressed negative opinions, listed in four main categories: the worry that patients would be harmed and upset by the information received; that patients would misinterpret the content and therefore more time would be required to explain the technical terms; the timing of the release of information which might be inappropriate; and that access might expose doctors as uncertain and prone to error (Fisher and Britten 1993).

Another study involved distributed questionnaires in an academic hospital to evaluate the response to a trial of giving patients daily progress notes (Weinert 2017). They reported that a range of 74 to 86% of patients responded favourably as it improved their understanding and feeling of control, while 9–28% of healthcare providers felt that they were more careful with what they wrote in the progress notes. However, 72% of the healthcare providers said the contrary. As this was based in one centre with a small number of participants (75 patients and 12 providers), this study is limited due to its lack of external validity (Weinert 2017). Thus, the doctors reported taking longer and were cautious with the style of documentation to address how a patient or family could react.

A separate study found that patients’ reasons for requesting medical records included a desire for control, taking responsibility and looking for inaccuracies (Wibe et al. 2011). The themes were having a sense of control, the patient feeling co-responsible for their own health and a lack of trust in healthcare professionals to look out for their best interests. Taking responsibility gives patients a sense of control and examining accuracy may lead to the discovery of some errors which can be rectified. However, reading the records caused patients to feel undervalued and stigmatised due to their lifestyle problems (Vermeir et al. 2017). It gave them the impression that they were not valued as individuals. As indicated by two sub-themes, this sentiment arose primarily when the patients thought that healthcare providers had undervalued them, displayed their preconceptions, or had narrow a perspective on their condition. When reading the record, patients also felt that doctors had wrongly attributed certain conditions to stigmatising lifestyle problems that the patients claimed not to have or indeed prioritise (Wibe et al. 2011).

A meta-analysis of 30 studies in the UK reported that patient access was unlikely to cause harm and generally had modest benefits, especially on doctor-patient communication and increased patient satisfaction (Ross and Lin 2003). Another meta-analysis of 18 studies reported a welcome step for patient-centred care to facilitate verbal communication in a doctor-patient relationship but that simply allowing full access without further explanation or summary was insufficient for the patient’s full understanding of the condition (D’Costa et al. 2020). This probably relates to the language or medical terms that are unfamiliar to the patient.

On the other hand, an earlier study was conducted in London where patients and consultants were selected for interviews and the consultants who criticised patient access claimed that patients would be perplexed with the information, as they were unfamiliar with the medical terminology and language (Britten et al. 1991). They also said that they would have to redact any material that has any degree of subjective views as the notes were written for doctors to establish a private dialogue amongst themselves. Lee et al. conducted a study of patient notes to identify barriers to patient access to medical records and found that those which confused patients, generated offence or had an impact on perceptions and professionalism were those that used medical words with judgemental connotations, errors and the use of jargon (Lee et al. 2017).

While a full appraisal of these studies is outside the scope of this article, the studies provide evidence that there are two perspectives on patient access—doctors and patients—who have different expectations and beliefs, and each occupy different roles that drive a successful doctor-patient relationship. Interestingly, these studies are arguably inferred from social desirability and self-perception as opposed to using more objective outcomes that concur with quantifiable benefits to support the notion of utilitarianism.

These outcomes from the patient’s perspective seemingly express true and experiential benefits such as improvement in patient satisfaction, control and communication and negative effects such as feeling devalued or stigmatisation for lifestyle choices. The patients self-reported and experienced desirable and undesirable outcomes which are thus ‘true or experiential’ benefits in which an event has occurred and been experienced. Interestingly, the harms outlined in these studies such as inappropriate information, inappropriate timing, increased confusion and potential distress are mainly generated from the doctor’s perspective, which can be considered theoretical and non-experiential. There is a pattern here in which the doctors continue to decide on behalf of their patients what is best for them. While this can be considered paternalistic, we can also claim that the doctor’s duty is to advocate for patients and therefore concur with the conclusions of these studies that doctors are concerned about unrestricted patient access and the possible negative effects.

## Utilitarian Approach

The empirical findings underscore the need to evaluate access to medical records from both ethical and practical perspectives. In utilitarianism, decisions are made based on an outcome that will produce the greatest amount of benefit for the greatest number of individuals (Amer 2019). Therefore, the morality of an intervention is determined by its consequences (Mack 2004). However, while the aim is the maximum benefit, the approach could lead to undesirable effects for some individuals. While the evidence is not exhaustive, the utilitarian approach is guided by the calculated benefits and harms of improving patient access. Despite limited evidence, we could infer from the perspectives of doctors and patients that it produces clear overall ‘experiential’ benefits for patients. To embrace the utilitarian perspective of wider access for patients competent to play a role in decision-making who request their medical records, we can predict overall benefits as reported in the literature such as facilitating communication, improving understanding, breaking the barrier between the doctor/patient relationship, feeling more in control of healthcare decisions and identifying errors and inaccuracies in the record (Delbanco et al. 2010). The experiential benefits reported by patients are stronger than the inferential negative outcomes, or harms, contemplated by their physician counterparts and therefore justify wider patient access. As importantly, given the negative effects of wider patient access as derived from the doctor’s perspective including some self-experiential concerns such as a change in the practice of documentation, more time is required to explain to patients the content in the medical records and liability issues. However, these concerns raised by doctors in respect of wider access can be addressed with adequate resources and training as well as increasing capacity in healthcare communication.

Thus, the concerns raised by healthcare providers seem to be largely based on theoretical risks rather than actual negative outcomes. While providers express worries about the potential misinterpretation of medical records or emotional distress, evidence indicates that these risks are relatively minor compared to the tangible benefits experienced by patients. Increased patient satisfaction, improved decision-making and a greater sense of control over one’s health significantly outweigh the theoretical harms, further supporting the utilitarian argument for wider access to medical records. Furthermore, patients’ desires to verify the accuracy of their records and ensure that their treatment is based on correct information are consistent with the utilitarian emphasis on maximising the benefits to individuals by empowering them to make informed decisions. Allowing access can help patients identify errors in their medical records, which could ultimately improve their healthcare outcomes.

Furthermore, legislation and national guidelines in various countries mandate patient access to medical records primarily to respect patient autonomy. While autonomy is not an element of utilitarian theory, its value is essential to the individual because it determines their ability to act according to their own interests and values. It is thus a utility as it drives one’s involvement and innovation to achieve what is perceived as positive and beneficial by the individual, which subsequently extends to the institution, community and society (Cheverie 2015). Respect for patient autonomy has become a primary tenet of medical ethics as it is a desirable outcome for patients to have their values and beliefs involved in the self-determining process of clinical decision-making (Coggon and Miola 2011).

## Deontological Approach

While there is an argument to justify wider access using the utilitarian theory, the evidence to support the benefits of this action is limited. Kant’s deontological theory provides a supplementary support to utilitarianism, to justify wider access. Although deontological approaches are usually described as being opposite, unlike utilitarianism theory, which focuses on outcome, deontology focuses on ethics where the morality of an action depends on its nature, irrespective of its consequences (Mandal et al. 2016). This article will determine whether the action of restricting or non-widening patient access following a patient request is justified.

In respect of wider patient access, this theory hypothesises that the ‘right’ thing to do is to allow patients to have access to their own information in medical records which must be prioritised as ‘good’. Therefore, restricting patient access is considered ‘wrong’. This theory also emphasises the element of autonomy which is part of the moral obligation for a certain action. Allowing automatic access to medical records when a patient requests it is always the ‘right’ thing to do. The essence of the argument to access medical records relates to the information contained within these records, and this includes information on the patients themselves. The Oviedo Convention (Council of Europe 1997), Article 10(2), states that:

Everyone is entitled to know any information collected about his health. However, the wishes of an individual not to be so informed shall be observed.

The patient’s right to information can be framed as an important element of the doctor’s obligation to their patient, as part of acting for the patient’s benefit and respecting their autonomy as stated in the Hippocratic Oath.^2^ Therefore, withholding information from a competent patient, especially in relation to the patient themselves, is morally unjustified and infringes on the patient’s right to information and autonomy.

Patient access to medical records is an act of or exercise in truth-seeking and can be considered an act of accessing the truth about oneself because truth-telling in a clinical consultation is ‘an act of sharing one’s knowledge and the limitations of one’s knowledge with accuracy and with sensitivity to the clinical impact of the disclosure’ (Roberts and Dyer 2003). Truth-telling is part of a doctor’s duty to be transparent and honest in the delivery of information. One study defied the speculation among physicians that most patients do not want to hear the truth (Sullivan et al. 2001). The vast majority of patient participants said they did want to know the truth about their health and that doctors had a duty to be honest with them, particularly when they were diagnosed with a life-threatening illness (Sullivan et al. 2001). Another study discovered that a lack of openness makes patients feel resentful and that distrust is a strong motivator for patients to request access to medical records (Wibe et al. 2011). Since access to medical records is part of truth-seeking, refusing a patient’s request to see their records is morally wrong. As a truth-seeking exercise, deontological theory maintains that the action of automatic granting of a patient’s request for access is morally good and withholding the truth is ethically unacceptable. According to this theory, medical records should be an accurate and truthful account of a consultation, and withholding this truth must be considered immoral.

There are implications for wider access as the doctor’s autonomy is eroded as the creation and content of medical records can be considered their intellectual property according to the MMC (Malaysian Medical Council 2006). According to deontology, consequences such as this should never matter, and as a rights-based theory, the ‘good’ should always prevail. However, the practice of truth-seeking and truth-telling in healthcare is complicated as doctors deal with individuals, usually at their most vulnerable, with potentially undesirable effects. While deontology proponents claim that the consequence should not matter, the limitation of this theory is that it does not address a conflict that may arise between two moral individuals, in this case, the doctor and patient. While this theory is inadequate to support and facilitate the real-time and real-life dynamics of a complex doctor-patient relationship, it provides support to the overall discussion that restricting access is not ethically justifiable.

## Malaysian Context: Social, Legal and Ethical Considerations

The legal and ethical landscape surrounding patient access to medical records in Malaysia is occupied with contradictions, particularly with the imbalance of power between healthcare providers and patients (Academy of Medicine Malaysia 2016). While the legal framework acknowledges patients’ rights to access their medical records, it fails to enforce this right consistently. This is compounded by a complex ethical debate, where principles like autonomy and truth-telling conflict with professional practices that often prioritise paternalism and confidentiality over transparency. This tension between legal limitations and ethical ideals sets the stage for a deeper exploration of moral theories that can justify a shift toward wider patient access.

The regulatory framework governing patient access to medical records in Malaysia is shaped by several key social, legal and ethical factors. These elements not only inform the current practices but also underline the complexities and contradictions within the healthcare system, influencing both patient rights and healthcare providers’ obligations. This disparity, combined with cultural norms around respect for authority and doctors (Lee et al. 2022), may influence how patients approach the issue of accessing their medical records. The prevailing culture, where patients tend to trust doctors as authorities, may contribute to their passive acceptance of limited access to their medical records. This trust can, however, work both ways, either facilitating cooperation with healthcare providers or enabling reluctance to challenge the status quo. Thus, the Malaysian social context complicates the pursuit of wider patient access to medical records, as the cultural respect for medical professionals often results in delayed or limited engagement with regulations that advocate for access.

The legal framework is fragmented, with inconsistencies in the application of guidelines for accessing medical records, as noted in the MMC Guidelines of Medical Records and Medical Reports (Malaysian Medical Council 2006), Guidelines for the Management of Patient’s Medical Records for Hospital and Medical Institutions (Ministry of Health 2010) and the Good Medical Practice Guidelines (Malaysian Medical Council 2019). These documents collectively affirm that while patients have a moral and ethical right to access their medical records, the legal practice falls short of providing an automatic or straightforward right. The legal system primarily enforces access through court orders, a process that often delays or obstructs immediate access, leading to additional costs and emotional distress for patients. This gap in legal recognition further complicates the situation, reinforcing the notion that patients must rely on litigation or third-party involvement to obtain access to their medical records.

The Malaysian legal framework also places significant control in the hands of healthcare providers, often leaving patients with limited recourse unless a court intervenes. This imbalance of power is evident in cases such as *Nurul Husna*,^3^ where the court acknowledged the patient’s right to access medical records but found that the medical provider still holds significant discretion over when and how these records are released. Such legal inconsistencies create a barrier to universal access and reflect a system that, despite acknowledging patients’ rights, limits their practical application.

The MMC Guidelines assert that healthcare providers are responsible for protecting patient information, balancing this with a moral obligation to share relevant information with patients (Malaysian Medical Council 2006). However, the ambiguity surrounding the ethical ownership of medical records where information is ethically shared but physically owned by healthcare providers creates a tension between transparency and the perceived paternalism of withholding information for patients’ well-being. This tension often leads refusal of medical records, especially in cases where the release of information could potentially result in legal challenges or reputational damage for healthcare providers.

Further, the ethical principle of respect for autonomy is often cited in the context of patient rights to information. However, despite moral assertions regarding ownership, these ethical principles are not always reflected in the regulatory framework. The MMC’s ambiguous stance on patient access, where access is conditional and not automatic, undermines the ethical commitment to respect patient autonomy and self-determination.

## Discussion

The issue of patient access to medical records in Malaysia is deeply intertwined with social, legal and ethical factors, each of which presents challenges to the realisation of a more transparent and patient-centric healthcare system. The existing regulations and practices reflect a tension between safeguarding healthcare providers and upholding patient rights. This delicate balance, coupled with cultural respect for medical authority and inconsistent legal frameworks, creates significant barriers for patients seeking access to their health information. The cultural deference to medical professionals further complicates the situation, as patients often accept the limited access to their medical records without questioning it. Additionally, the legal framework, while recognising the moral right of patients to access their medical records, fails to provide a clear or automatic right, forcing patients to rely on legal action or third-party intervention. To address this issue, it is important to apply ethical theories such as utilitarianism and deontology to evaluate whether Malaysia should widen patient access to medical records.

While both utilitarianism and deontology offer compelling justifications for expanding patient access to medical records, the Malaysian legal system may still present challenges in aligning these moral imperatives with practical reality. The current legal framework marked by its reliance on court orders and the discretion of healthcare providers often obstructs timely access, resulting in delays, additional costs and emotional distress for patients.^4^ This legal ambiguity reflects a deeper tension within the healthcare system, where the rights of patients and the discretion of healthcare providers are not clearly delineated. As discussed, a case like *Nurul Husna* shows that the legal system acknowledges the right to access but places significant limitations on how and when patients can exercise this right. The healthcare system, therefore, finds itself in a bind: while legal recognition of access is essential, it is often not enacted swiftly or clearly, resulting in unequal and inconsistent treatment of patients.

To bridge the gap between ethical theory and legal practice, there is a critical need for reform in Malaysia’s healthcare regulations. From an ethical standpoint, both utilitarian and deontological theories support the idea that patient access to medical records should be expanded. Reform could take the form of clearer legal standards that automatically grant patients the right to access their medical records, without the need for court orders or third-party intervention. Additionally, healthcare providers should be encouraged to adopt more transparent practices, which respect patient autonomy and facilitate easier access to information. Finally, cultural attitudes toward medical authority may need to shift, empowering patients to be more proactive in requesting their records and engaging in discussions about their healthcare.

The utilitarian approach, which proposes that the right action leads to the most beneficial consequences, is supported by empirical evidence suggesting that wider patient access mainly benefits patients. This aligns with the argument that access promotes respect for patient autonomy. Deontological proponents claim that if access to medical records is considered a truth-seeking exercise, then the act of restricting patient access should always be considered immoral, despite the consequences. ‘Truth’ is another important element discussed in the deontology section, to which the principles of respect for autonomy are linked. The moral argument in favour of truth-telling is an obligation of fidelity and the need for trust in the doctor-patient relationship. Trust is the essential quality that drives a good doctor-patient relationship which could not be built on untruthfulness (Stirrat and Gill 2005). Untruthfulness or telling lies is the extreme negative end of the spectrum of doctor-patient communication.

In fact, better communication and truth-telling in an exercise where truth is sought by the patient for whatever reason, these practices should be considered an integral part of the doctor-patient relationship, especially if trust is considered a central tenet of the relationship. These three lines of reasoning raise the question of whether therapeutic privilege, which drives the argument against widening patient access in this article, is obscured and unjustified.

## Conclusion

This paper has articulated the ethical justification for wider patient access to medical records using both utilitarian and deontological theories. I have built up views, reasonings and justifications using these two important moral theories. The argument on both utilitarian and deontology supports the notion that Malaysia should widen patient access. However, acknowledging the different views of doctors and patients is of paramount importance as it helps to reform and align the ethical and legal reasoning to widen patient access. The different views and understanding of how this debate interacts within this complex regulatory space act as a temper in finalising the situation and environment, legally and ethically, in making wider patient access a reality.

The convergence of these two ethical frameworks highlights the need for reform in Malaysia’s healthcare system to align legal and ethical reasoning with the benefits of wider patient access. Recognising and addressing the differing perspectives of doctors and patients is crucial in making this reform a reality. Ultimately, this paper advocates for the implementation of policies that facilitate greater patient access to medical records, ensuring that ethical principles of autonomy, truth-telling and utilitarian benefit are upheld in the healthcare setting.

## Footnotes

1 Nurul Husna Muhammad Hafiz & Anor v. Kerajaan Malaysia & Ors [2015] 1 CLJ 825.

2 R v Mid Glamorgan Family Health Services Authority ex parte Martin [1995] 1 All ER 356.

3 Nurul Husna Muhammad Hafiz & Anor v. Kerajaan Malaysia & Ors [2015] 1 CLJ 825.

4 Nurul Husna Muhammad Hafiz & Anor v. Kerajaan Malaysia & Ors [2015] 1 CLJ 825.
